# Implications of Dietary Intake and Eating Behaviors for People with Serious Mental Illness: A Qualitative Study

**DOI:** 10.3390/nu14132616

**Published:** 2022-06-24

**Authors:** Annabel S. Mueller-Stierlin, Sebastian Cornet, Anna Peisser, Selina Jaeckle, Jutta Lehle, Sabrina Moerkl, Scott B. Teasdale

**Affiliations:** 1 Department of Psychiatry and Psychotherapy II, Ulm University, 89070 Ulm, Germany; annabel.mueller-stierlin@uni-ulm.de (A.S.M.-S.); sebastian.cornet@uni-ulm.de (S.C.); selina.jaeckle@uni-ulm.de (S.J.); jutta.lehle@uni-ulm.de (J.L.); 2 Department of Psychiatry and Psychotherapeutic Medicine, Medical University of Graz, 8036 Graz, Austria; anna.peisser@stud.medunigraz.at; 3 Discipline of Psychiatry and Mental Health, University of New South Wales (UNSW) Sydney, Sydney, NSW 2052, Australia; s.teasdale@unsw.edu.au; 4 Mindgardens Neuroscience Network, Sydney, NSW 2052, Australia

**Keywords:** mental illness, depression, psychosis, bipolar, diet, nutrition, qualitative study, implications

## Abstract

The impact of poor diet quality and nutritional inadequacies on mental health and mental illness has recently gained considerable attention in science. As the opinions and experiences of people living with serious mental illness on dietary issues are unknown, we aimed to understand the role of nutrition in a biopsychosocial approach. In total, 28 semi-structured interviews were conducted with people living with serious mental illness (SMI) in Australia, Germany and Austria, and a generic thematic analysis approach was applied. Four positive (positive effects on the body and mind, therapeutic effects in treating somatic illnesses, pleasure and opportunity for self-efficacy) and three negative (impairment related to mental illness and its treatment, perceived stigma and negative effects on the body and mind) implications of diet were identified. A key issue for most of the participants was the mental burden arising from their body weight. This might indicate that negative implications, such as guilt and stigma, were of primary importance for people with SMI when talking about their dietary behavior. In conclusion, diet-related support is urgently needed for people with SMI. However, especially participants from Germany and Austria reported that this is not yet widely available in mental health settings, leading to hopelessness and resignation.

## 1. Introduction

The health disparities experienced by people living with serious mental illness (SMI), such as schizophrenia and related psychoses, bipolar disorder and major depressive disorder, are well described [1]. Physical health disparities, such as cardiovascular disease and diabetes, are the major drivers of the 13–15-year mortality gap in people with SMI compared to the general population [2], with two-thirds of deaths attributable to natural causes [3]. The drivers of these physical health disparities are multifactorial, relating to stigma, structural discrimination and diagnostic overshadowing [4,5]; service disconnect and poor access [6]; characteristics of the illness and medication side effects [1,7]; high rates of smoking and substance use [8,9,10]; high levels of sedentary behavior [11]; and excessive dietary intake of poor quality [12].

A 2019 systematic review and meta-analysis included 58 studies that reported on the dietary intake of people with schizophrenia and related psychoses and bipolar disorder and found higher caloric and sodium intakes, less healthful dietary patterns and lower diet quality compared to people without a mental illness or the general population [12].

The reasons for the excessive and less nutritious dietary intakes often consumed by people with SMI are multifaceted and intertwined. Among others, treatment for SMI commonly consists of psychotropic medication—particularly antipsychotic and mood stabilizer medication—which can increase appetite [13]. Impaired executive functioning—commonly associated with psychotic illnesses—facilitates disinhibition and complicates restrained eating [14]. Disordered eating behaviors, such as binge eating, and unhealthy eating styles linked to the state of mental health, such as emotional eating, are highly prevalent in people living with SMI [15]. Further, people with SMI experience food insecurity more frequently than people without SMI [OR = 2.7] [16], with food insecurity linked to less healthful dietary intake [17].

More recently, the impact of poor diet quality and nutritional inadequacies on mental health and mental illness has gained considerable attention [18]. Diet quality is inversely associated with depressive symptoms and incident depression [19], with a whole-of-diet approach being effective in reducing depressive symptoms [20,21] and cost-effective in terms of QALYs [22,23].

The opinions and experiences of people with SMI are critical to progressing the role of nutrition for both physical health and mental health. Thirteen qualitative studies were identified in the published literature by the research team [24,25,26,27,28,29,30,31,32,33,34,35,36]. Most studies have focused on physical health issues: three studies explored people’s experiences of weight gain secondary to an SMI diagnosis and commencement of psychotropic medication [24,25,26], and six studies explored people’s experiences of physical healthcare in mental health services (including lifestyle and behavior change interventions) [30,31,32,33,34,35]. Only four studies had a more targeted aim of understanding the experiences of nutrition in people with SMI [27,28,29,36]; however, the psychosocial implications of diet seem to be rarely explored. Nevertheless, the biopsychosocial model is widely accepted as indispensable to understanding mental health. This model assumes that an interdependent relationship exists between biological, psychological and social factors, which are involved in all aspects of mental health. 

To inform a broader research program [37], we undertook a qualitative study in people with SMI across three countries, with the aim of understanding the role of nutrition in a biopsychosocial approach.

## 2. Materials and Methods

### 2.1. Design

This study involved semi-structured interviews with thematic analysis across three mental health service sites: Ulm, Germany; Graz, Austria; and Sydney, Australia. Interviews were conducted between October 2019 and September 2020. Ethics committee approval was obtained from each site: Ethics Committees of University of Ulm (protocol code 414/19, 28 January 2020), Medical University of Graz (protocol code 32-178 ex 19/20, 20 January 2020) and South Eastern Sydney Local Health District (SESLHD) (2019/ETH12620, 14 November 2019). 

### 2.2. Participants

Participants were recruited from three mental health services: the regional district hospital in Günzburg, Germany; Department for Psychiatry and Psychotherapeutic Medicine, Medical University of Graz, Graz, Austria; and SESLHD Mental Health Service, Australia. Participants were recruited using onsite advertisements and word-of-mouth via mental health clinicians and researchers. Inclusion criteria were: (i) able to provide consent, (ii) diagnosis of an SMI (schizophrenia and related psychotic disorders, first-episode psychosis, bipolar disorder or major depressive disorder), (iii) currently prescribed psychotropic medication and (iv) aged 18–60. Exclusion criteria were: (i) acute phase of mental illness, (ii) risk of adverse mental state, distress or discomfort as a result of participating in the interviews or (iii) current diagnosis of an eating disorder (anorexia nervosa or bulimia nervosa).

The target number of participants was 8–12 per site to reach anticipated thematic saturation, allowing for site-specific exploration, based on previous qualitative studies in people with SMI [35].

### 2.3. Procedure

Willing participants were enrolled in the study and were provided financial compensation for their time at study sites in Günzburg and Sydney. Each participant provided informed, written and verbal consent prior to commencing the semi-structured interview. The interview duration was set at 30 min, with a maximum duration of 45 min. Interviews were conducted by a member of the research team and recorded using a voice recording device. Interviews at the Sydney site were conducted in October 2019 in a face-to-face manner within an onsite consulting room at the mental health service. Interviews at the Ulm site were conducted in April 2020 using a mixture of telephone calls (*n* = 6) and web-based video conferencing (*n* = 2). Interviews at the Graz site were conducted between March and September 2020 using a face-to-face method onsite in a consulting room (*n* = 4) and via web-based video conferencing (*n* = 4). 

Interviews followed a semi-structured interview schedule (see Supplementary File S1). The interview schedule was created in English and then underwent forward-backward translation to create a German version. Each site used a different interviewer, and each interviewer had a different level of experience in conducting qualitative interviews. The interviewers engaged in a single training session to establish general consistency in the interview method. The interviewer clarified the intended meaning for two key terms: (i) diet referred to food choices, and (ii) eating behaviors referred to aspects such as appetite, speed of eating, structure of meals, emotional and binge eating, and overnight eating. Interview questions related to challenges and barriers to healthier food choices; experience of adverse eating behaviors; impact of mental illness and/or psychotropic medication on food choices and eating behaviors; impact of food choices on mental health; and what support for dietary intake/eating behaviors the participant had been offered/received via mental health services. Interview questions were asked as open-ended questions with the aim of the interviewer speaking as minimally as possible outside of asking the question. Prompts and question clarifications were provided by the interviewer where appropriate. Interviewers did not rephrase participant statements in their own words to avoid potentially changing the meaning and only provided example answers in cases where the participant was not understanding and requested support. Participants were given the opportunity to add to, retract or alter any of their responses immediately prior to concluding the interview. 

### 2.4. Thematic Analysis

The audio recordings underwent verbatim transcription. Identifying information was removed from transcripts, and pseudonyms were used to preserve anonymity.

**Figure 1:**
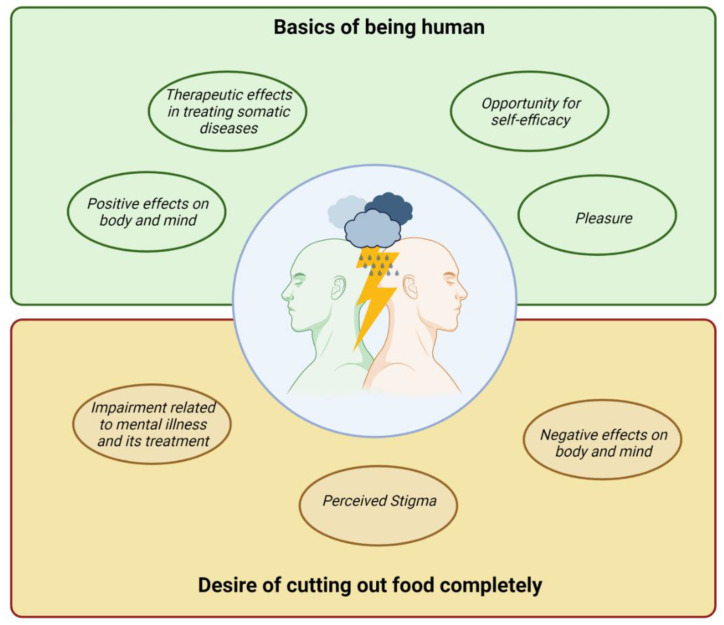
Thematic presentation of subjective implications of diet for people with mental illness. This figure was created with BioRender.com (accessed on 30 May 2022).

A generic thematic analysis approach was applied [38], led by AMS, SC and AP, who are fluent in the German language and fully proficient in the English language. After familiarizing themselves with the data, two researchers (AMS and SC) independently coded the data from German interviews (Ulm) by using MAXQDA 2020 (VERBI Software, Berlin, Germany) qualitative data analysis software. SC collated codes into potential themes and discussed them with AMS. Discrepancies in coding between the two investigators were resolved through discussion. Based on these initial themes, the same researchers (AMS and SC) continued similarly with the data from Australian interviews (Sydney), and subsequently, two researchers (SC and AP) replicated this process for the data from Austrian interviews (Graz). Emergent themes were reviewed and refined in relation to the coded extracts and the entire data set by all three researchers (SC, AMS and AP). Theme names and definitions were discussed among all authors, and a thematic map (see Figure 1) was created. To prepare the report of findings, compelling extract examples were selected and forward-backward translated, if collected in German.

## 3. Results

### 3.1. Study Population

Between November 2019 and September 2020, a total of 28 participants with SMI were recruited for semi-structured interviews. The breakdown of participants per site was: Australia (*N* = 12), Germany (*N* = 8) and Austria (*N* = 8). 

The age of participants ranged from 20 to 63 years, and 17 (61%) of those participants were female. The majority of participants were obese according to the WHO body mass index criteria (*n* = 16, 57%), with a mean BMI of 31.3 kg/m^2^. Sixteen (57%) participants reported having schizophrenia or related psychoses, 18 (64%) participants reported experiencing an affective disorder and (as defined in the inclusion criteria) all participants reported taking psychotropic medication (see Table 1).

### 3.2. Qualitative Findings

Participants’ experiences, beliefs and attitudes towards diet and eating behavior varied greatly, from very negative perceptions to positive perceptions, leading to an inner conflict. Four positive and three negative implications were identified after an in-depth analysis of the participants’ interviews. These themes are presented in Figure 1.

#### 3.2.1. Positive Implications of Diet

Nutrition is a fundamental need, and therefore, “*food, drinks, sexuality*” belong to the “*basics of being human*” (Ulm01, male). A proper diet is perceived as a prerequisite for health and well-being, clearly illustrated by the analogy of one participant: “*It’s like a car isn’t it, you put the wrong fuel in, doesn’t run properly, it might not run at all, it’s gonna break down*” (Syd08, female).

##### Positive Effects on Body and Mind

Participants recognized benefits to the body and mind when eating healthy food. Next to the taste, the expected effects on physical and mental well-being were important factors in choosing food considered to be healthy. Some examples are provided:

“*If I eat things that taste good to me, but are also generally considered healthy, then this has a positive effect for my mental well-being, I feel better, I have more energy and my digestion, for example, also gets better. So, it’s very important to me that my food tastes good and that it’s also good for my body. And [good] for the soul, yes*” (Ulm04, female).

“*Right when I eat healthy foods, which are healthy for my body, my mind just naturally benefits*” (Syd05, female).

“*Well, it’s already better, I now really eat a proper full meal with a salad and such and then I feel relatively long satiated and then that is just everything (…) Yes, one just feels better this way*” (Ulm03, female).

“*Fish in particular is a brain food, and I eat a lot of it. It helps me to focus on the issues, helps to concentrate. It’s also enjoyable, and healthy as well, good for your body*” (Syd08, female).

However, our interviews also show that some participants intentionally choose to eat some unhealthy food to do something good for themselves, at least temporarily. For example, chocolate and soft drinks are expected to have positive effects on mood: “*And when I eat the chocolate it feels relaxing*” (Syd04, male), or “*I reckon that if you’re drinking a lot of soft drinks, you feel tired, you get a boost*” (Syd04, male). In this way, the consumption of unhealthy food was partly justified with specific needs: “*I simply ate sweets a lot. Well, I felt like my nerves needed that*” (Graz05, female), or “*Cause I feel weak and tired, and then I need energy and I eat junk*” (Syd04, male). 

##### Therapeutic Effects in Treating Somatic Illnesses

Several participants reported eating well with the aim of supporting the treatment of physical comorbidities. Thus, three participants from Sydney reported changes in dietary behavior to reverse diabetes type 2 (Syd01, male; Syd02, female; and Syd11, female). One participant claimed that she had overcome cancer by adopting a ketogenic diet (Syd11, female).

##### Pleasure

One participant emphasized how important it is for him to mindfully enjoy food “*I just take my time. I enjoy eating, so now it is more than satisfying hunger for me. [*…*]. Just pleasurable*” (Ulm01, male). He explained: “*Food results in delight and a good mood, so yes*” (Ulm01, male). This participant also reported that he himself had experienced a strong change in his food-related attitudes. He used to eat a lot of fast food, which was filling but “*certainly not healthy emotionally nor organically*” (Ulm01, male). He described situations when he was “*just being in the kitchen, without a plate, just standing like that, open package, slap it on, bam. Them I am full so to speak, but not happy*” (Ulm01, male). Now, he said, it is a completely different living experience for him, and he fully enjoys the food. Other participants highlighted the pleasure associated with eating and described themselves as “*epicurean*” (Ulm05, female) and “*gourmet*” (Graz07, female). 

##### Opportunity for Self-Efficacy

In the context of dietary behavior and food choices, participants reported that they had experienced how their own actions can contribute to improving their own health and well-being. These personal experiences of control and success build self-efficacy. 

Two participants described the impact of eating health food and cooking for themselves as follows: “*It certainly has an impact. When you eat good food, your mood is certainly quite different from when you eat randomly. I am relatively sure that this has an effect. I mean, these are not major ones, so that if I just eat healthy food, my mood is always on top, of course not, but it does contribute to the fact that it is like this*” (Graz01, male), and “*mentally you feel good about yourself cause you’ve eaten healthy, (…) and like it’s a bonus if you cook it yourself, like have that satisfaction that I’ve cooked something for myself, I’ve, I’ve eaten it and it’s healthy for you and in a sense, it puts you in a good mood*” (Syd03, male). Another participant described that she sees nutrition as an opportunity to improve her health. “*I would say that it would make me feel significantly better, I suppose, feeling like oh I am actually doing something pretty good for my body*” (Syd06, female).

Ultimately, the experience of the positive effects of a healthy diet can also lead to further positive enhancement of dietary behaviors: “*motivation to eat healthier food just comes naturally because I gain not also pleasure from taste, but I notice that physiological improvements in my body and mind after eating healthier foods, which just naturally drives my eat more and more healthy foods*” (Syd05, female).

In addition, participants also see their eating practices as a way to cope with mental health issues. For example, one participant reported that she hardly eats anything during mental crises to make herself feel that she still has control over her life (Ulm06, female). For others, eating seems to be a coping mechanism, which distracts from other worries and loneliness in the short term. “*Yes, with certain emotions, yes, there is often a feeling of hunger or that I get an appetite for sweets or greasy things or bread or pasta. […] For example, when I’m angry or worried or afraid, and I don’t necessarily have someone to discuss these feelings with right away. So, when I am simply alone with these feelings and have to see how I can cope with them on my own*” (Ulm04, female), or “*sometimes you eat there because you’re bored or your lonely, or you think it’s, when you eat at you feel like you’re in company. If you feel a bit lonely you eat, you feel like there’s someone extra with you by eating*” (Syd4, male). One participant even sees her eating behavior as an option for self-harm. “*So, I think that was also a little bit of maybe self-harming behavior, I just didn’t want to do that anymore. I didn’t want to eat anymore somehow*” (Ulm03, female).

#### 3.2.2. Negative Implications of Diet

For some participants, eating is primarily a burden and not a source of pleasure. This goes so far that one participant even said, “*Well, it would be easier for me if you could cut out food completely*” (Ulm07, female).

##### Negative Effects on Body and Mind

Some participants identified physiological effects of an unfavorable diet. These include sleepiness, restlessness and feeling heavy. One participant described his challenges with portion control as follows: “*So, I notice that when I get hungry, I get really cranky, so I was always trying to pick fights when I was skinny manic, manic skinny. I was picking fights with everyone. (…) But when I’m too full I get way too sluggish, I get super sleepy. Yeah, I get slow*” (Syd12, male). 

Another participant described the “*vicious circle*” (Graz03, female) that results from these effects in combination with her mental illness (i.e., medication-related ravenous hunger attacks and lack of drive): “*And when I have these ravenous hunger attacks, then it’s not so great, I feel so heavy and sluggish. (*…*) Then that is counterproductive for my drive*” (Graz03, female). 

##### Impairment Related to Mental Illness and Its Treatment

Some participants reported that mental health impairments were mirrored in dietary behaviors, and thus, mental illness and diet were closely intertwined. The burdens associated with mental illness were thus also attributed to nutrition.

This is clearly illustrated by a quote from a patient with post-traumatic stress disorder who associated eating with oral sex because of her traumatic experiences. “*And for me, my eating behavior is also partly related to my trauma experiences, so for example I often find it difficult to eat something (*…*) That triggers me, so for example it reminds me of oral sex or something like that*” (Ulm06, female).

Other participants reported having developed addictive behaviors related to food in the course of their mental illness. For example, participants noted, “*suddenly I got addicted to eating*” (Syd12, male), or “*I just got the addiction, when the addictive behavior comes, I can’t say no anymore*” (Ulm07, female). In addition to the coping strategies already mentioned, the participants considered one reason for this to be the side effects of the medication.

In the end, some participants see the medication as the cause of dramatic weight gain, which, however, cannot be attributed exclusively to changes in dietary behavior, but whose biological background remains partly unexplained.

To be specific, some participants reported an average weight gain of about one kilogram per week during some periods of time (Graz05, female; Syd10, female; Ulm05, female). The weight fluctuation of one participant started at about 115 kg, followed by a strenuous weight reduction to 65 kg and then a relapsed increase to about 140 kg (Ulm07, female). Based on a discontinuation attempt, one participant was able to clearly link weight fluctuations to medication use: “*I also tried to stop taking the medication, with the approval of the attending specialist and with support, and I lost weight without any problems and did not go on a diet. So, I simply saw how much this medication somehow influences the body and then I... But it never worked without tablets, then I had to take psychotropic drugs again and then I put on weight again. So, I also saw that, yes, the medication has a very, very huge impact on my body*” (Ulm04, female). Another participant reinforced that the weight changes were clearly related to medication intake, but not to changes in their own dietary behaviors. “*Cause as soon as I was prescribed clozapine* [an atypical antipsychotic], *I didn’t think I would eat anything other than normal, but my weight would, I would just get bigger and bigger and bigger and like I would just balloon, like for no reason […] I didn’t eat any different, I didn’t, that’s probably why, I didn’t eat any different*” (Syd11, female). However, for another participant, the medication triggered food cravings, and these resulted in the weight gain (Graz02, female).

One participant reported that even psychiatrists are not sufficiently aware of the relationship between psychotropic drugs and weight changes, as “*All in all, neuroleptic or psychotropic drugs and obesity are not yet well known among doctors, especially what the effects could be*” (Ulm04, female).

The statements of some participants show the great mental burden that can occur when people gain weight. In some cases, these have led to strong fears*:* “*I have a panic-like fear of gaining weight*” (Ulm06, female), and “*I was so insanely afraid I would fall back into the old pattern*” (Ulm07, female). Some participants’ statements also reveal a kind of vicious circle, a downward spiral. Weight gain leads to negative feelings such as frustration and self-doubt, from which one distracts oneself by eating as a coping mechanism: “*And for me it was such a cycle. (*…*) When I put on weight, (*…*) and then you feel unwell. (*…*) And then you eat again out of frustration. (*…*) If you eat again, it gets worse. And then the frustration becomes even greater*” (Graz02, female). The unsuccessful attempt at weight management led to a depressive episode for one participant: “*Despite all of the effort, I have nothing else in mind other than this [to avoid weight gain] (*…*) and in the end I found myself, in my case, in deepest depression, because I was really at the bottom*” (Ulm07, female).

As a consequence, some participants described how they convulsively try to counteract further weight gain through changing lifestyle behaviors, such as physical activity (Ulm04, female; Syd06, female), as well as dietary behavior.

“*So, when I’m hungry and I don’t want the extra kilojoules, I just force down a cup of coffee and I manage to control my appetite. That’s how, I think how I’ve been maintaining my weight (…) We just want to eat all the time and it’s annoying me*” (Syd12, male).

“*Chocolate is not essential for life and that is now my first step, where I think to myself, alright, do not eat the first piece of chocolate to which I stick to very convulsively*” (Ulm07, female).

##### Perceived Stigma

Another stress factor is that people with overweight/obesity often experience the prejudices of other people as well as discrimination. This was also reported several times in the present interviews: “*people calling you pig and all this stuff*” (Syd04, male), and “*Sometimes I don’t like to go to the shopping center because people are watching me eating and calling me a pig*” (Syd04, male). One participant has already internalized negative assumptions about being overweight: “*I’m the fat one after all. The fat one who should keep her mouth shut (*…*) Being fat is unattractive, and people don’t like it and from that perspective I am powerless (*…*) everybody likes thin people. And I am a fat person who is being stared at and who constantly is devalued and who is put down and about whom one gossips, about whom one talks, whom one stares at and, above all, about whom one laughs*” (Ulm07, female). In the experience of some participants, partnerships are also fundamentally influenced by being overweight: “*it’s also about my husband, he knew me when I was 50 kilos [now 112 kg], of course he has no desire for me sexually*” (Ulm05, female). Another one stated: “*I feel I missed the boat to get a wife because I’m fat*” (Syd04, male).

Even mental health professionals seem to be prejudiced against people who are overweight/obese. This becomes clear in this statement from one participant talking about her psychiatrist: “*You can’t lump everyone together and say, yes, the people with mental illnesses, they all just sit on the sofa, eat chips and drink soft drinks or something. So, I indeed have often been accused of this, without having been asked in detail, what my nutrition is actually like*” (Ulm04, female). Another one said: “*Especially people with mental illnesses are often suspected that they just don’t want to take care of their diet and, as with most things, it’s just not that simple*” (Ulm06, female).

On the one hand, this perceived stigmatization further strengthens participants’ desire to reduce weight (e.g., also through dieting); on the other hand, however, it also leads to social withdrawal. “*I want to be alone; I don’t want to have to go anywhere; I don’t want to show myself with my perpetual weight and shape, with my overweight, and I just want my... I want... I am withdrawing*” (Ulm07, female).

#### 3.2.3. Inner Conflict Related to Diet

Most participants end up in confusion and with an inner conflict related to their eating behavior, with which they feel left alone.

##### Inner Conflict

The perceived inner conflict occurs, e.g., when the participant opts for sweets: “*It’s no good for my stomach but they said it’s good for your soul. I suppose that’s true isn’t it*” (Syd08, female). However, in these cases, some participants still see the possibility of compensating for this by adjusting their dietary behavior in the coming hours or days (Ulm04, female).

However, the inner conflict is much more burdensome when it extends to dietary behavior as a whole. Only a few participants had succeeded in adopting a dietary behavior that favors the positive implications of food. Most participants were still struggling with the negative implications but were not able to change their eating behavior accordingly. Participants reported that they “*theoretically already know how to eat healthy*” (Ulm06, female), but that all interventions “*had only a short success*” (Ulm07, female), as they “*just can’t put it into practice*” (Ulm05, female) or as they “*could not stick to the rules*” (Ulm07, female). One participant explained these challenges in adopting a healthy diet by her emotionality and her distraction through other worries: “*I just can’t control that then, with the emotionality and also because I always have in my mind what is tomorrow and what is coming up again.* […] *I seem to be blocked. I don’t know what’s going on in my brain... And I just have a lot going on [in my head]*” (Ulm05, female).

As a consequence, negative feelings (e.g., dissatisfaction and guilt) usually occur during or immediately after food intake. For instance, eating for pleasure has only a short-term positive effect on well-being, as dissatisfaction sets in shortly thereafter: “*But if I am thinking about it again, ‘that was too much’, ‘too much bread or carbs or pasta’, then I’m not satisfied after all*” (Ulm04, female), or “*when I start reaching out for that chocolate, I start feeling horrible for like the whole day*” (Syd06, female). In addition, there are strong feelings of guilt after unhealthy meals: “*I always feel guilty when I’ve eaten [profanity], like junk food or takeaway or whatever*” (Syd03, male). “*It’s not only that I feel like my stomach feels bloated, but I also feel sick, I would end up feeling pretty angry towards myself along the lines of like, why did you take that chocolate bar even though you said so yourself that you’re not going to take it, and I would just kind of feel guilty about myself*” (Syd06, female).

The conflicting power of the positive and negative implications of eating and the burden associated with this inner conflict was described by one participant: “*While I am eating it, I actually feel bad already because I think to myself, it is not doing me any good at all. I’m not getting anything out of it, it’s just making me fatter. So, it’s not really satisfying, but I still can’t stop and say, ‘no, I’m going to stop and somehow get it under control in a different way.’ But it’s still comforting somehow. It seems as if now it doesn’t matter; now I can stuff myself with it, but actually I then feel worse than before*” (Ulm06, female).

##### Missing Support

Clear differences between the interviews with participants in Australia and in Germany or Austria were observed in the care provided for nutrition-related needs. 

Some participants from Sydney had already taken part in the “Keeping the Body in Mind” program [35] and thus received support from a multidisciplinary team, which aims to prevent weight-related side effects of psychotropic drugs through lifestyle interventions. This support was evaluated very positively by the participants. 

This contrasts with the rather negative experiences of participants from Ulm, Germany, and Graz, Austria. They reported that diet was only addressed in mental healthcare when the patients themselves raised the issue (Ulm01, male; Ulm04, female; Graz02, female). Even then, participants felt that they were not being properly listened to. “*So, I think, first of all, it would be important to be listened to at all, that is, if you have the opportunity to say, hey, I have the problem, I have gained weight, that this may take place at all, this conversation [*…*] I would first of all wish that I am actually listened to with these problems*” (Ulm06, female). Although help was sought, one participant’s experience was that this did not bring about any improvement to her mental health: “*I went everywhere and tried to get … help somewhere, but nothing was available*” (Ulm07, female). This in turn brings feelings of hopelessness and resignation. Participants reported that psychiatrists do not take their complaints seriously or that they do not receive adequate support in this regard. 

The participants also complained that their doctors did not take their weight history into account when prescribing medication. “*I have also always pointed out to the doctors on time that I am gaining weight too quickly, but they have not acted on it*” (Ulm04, female). Some participants quoted their psychiatrists: “*it’s impossible to lose weight with the tablets*” (Ulm04, female), and “*with this medication you can dream of losing weight, nothing works at all*” (Ulm05, female). One problem is that this can partially lead to low compliance since the persons suffer severely from the weight gain. This inner conflict is also evident in the present interviews: “*Olanzapine [atypical antipsychotic] is so bad*” (Syd12, male), or “*My God, I have to take it, what else can I do, I cannot help it*” (Ulm05, female). 

In summary, it can be stated that, for many participants, weight changes have had negative implications for their mental as well as their physical health. In some cases, weight changes are considered a relevant cause of anxiety and depressive episodes or can intensify them. Statements of some participants give additional indications of a possible developing or already existing eating disorder. Considering these issues, it becomes increasingly understandable that some participants have mostly negative associations with food. 

## 4. Discussion

In this qualitative study, the experiences, beliefs and attitudes of 28 participants living with SMI regarding diet and eating behavior were investigated. Study participants described a strong inner conflict regarding their dietary behavior. Positive implications, including the therapeutic effects of nutrition on the body and mind, as well as the treatment of somatic illnesses, pleasure and self-efficacy, were described. However, patients cited negative effects on the body and mind, eating issues related to their illness such as traumatic experiences, the negative consequences of treatment and perceived stigma. 

Participants reported that their dietary choices and eating behavior had a bidirectional relationship with both their physical and mental health and highlighted the advantages of eating regular healthy meals in particular. This is consistent with the broader literature. A meta-analysis of observational studies found that adherence to a healthy/traditional dietary pattern, e.g., Mediterranean or avoiding a pro-inflammatory diet, protects against incident depression, with 33% and 24% reduction, respectively [19,39], and RCTs have demonstrated that dietary improvement reduces depressive symptoms, particularly in people with clinical depression [20]. Irregular eating patterns can be associated with mood changes [40], while eating regularly and choosing foods with a slow energy release may help to stabilize mood. Eating regular meals is also inversely associated with insulin resistance and metabolic syndrome, a common comorbidity in people with SMI [41]. At the same time, skipping meals has been associated with depression, problems with sleep, obesity and suicidal ideation [42,43]. 

However, people with SMI may not adequately distinguish between the long-term and short-term effects of diet and appear to use food as a coping mechanism to distract themselves from worries and fears in the short term. This can lead to low mood [39], creating a cycle whereby low mood then drives cravings for highly palatable foods high in sugar, salt and fat to generate short-term pleasure and reward [44,45]. Further, the perceptions of our study participants who stated that “*chocolate feels relaxing*” and “*I felt my nerves needed that [sweets]*” underline a possible serotonin deficiency as a reason for sugar cravings, as a carbohydrate-rich diet triggers an insulin response and increases the bioavailability of the serotonin precursor tryptophan in the brain [46]. In particular, carbohydrate craving was associated with tryptophan breakdown in patients with bipolar disorder [47]. In addition, long-term high sugar consumption has adverse effects on mental health [48]. Cravings may also be associated with certain nutrient deficiencies due to qualitative but not quantitative malnourishment (“*just being in the kitchen, without a plate, just standing like that, open package, slap it on, bam. Them I am full so to speak, but not happy*”) and emphasize the need for interventions of regular eating and mindful eating as a possibility to counteract food cravings in people with SMI [49]. Some study participants correctly identified the detrimental long-term effects of an unfavorable diet on mental health and cited symptoms such as sleepiness, restlessness and feeling heavy. Some pointed out that mental health problems (such as trauma) cause altered dietary behaviors (i.e., food avoidance, food addiction and cravings) and vice versa, leading to a vicious circle. Indeed, disordered eating behaviors, such as binge eating, and unhealthy eating styles, such as emotional eating, are highly prevalent in people living with SMI [15].

A key issue for most of the participants was the mental burden arising from their body weight. This problem often interferes with various areas of life and thus occupies a large part of everyday living. Body weight, stigma and mental health are interconnected, with increased body weight strongly associated with weight stigma and poorer mental health [50]. Many participants of our study reported experiences with weight-related discrimination by family members, foreigners and even mental health professionals. As a result of this, some participants reported social withdrawal and restrictive eating with subsequent weight cycling. Weight cycling can have negative psychological and behavioral implications with a greater risk for psychopathology [51]. For example, Quinn et al. (2020) showed that greater weight cycling is related to depressive symptoms, with stigma as a partial mediator, even after controlling for age, gender, education, income and body mass index [52]. Experimental studies point out that weight cycling causes pronounced stress on the metabolic system, with distinct fluctuations in blood pressure, heart rate, sympathetic activity and levels of lipids, glucose and insulin [53]. These factors contribute to metabolic comorbidities (such as diabetes mellitus and cardiovascular disease) as well as premature death [54], which are common in people living with SMI [2]. The participants of this study described medication as the cause of dramatic weight gain of about one kilogram per week. This is consistent with the broader literature, which has shown that particularly antipsychotics and mood stabilizers can increase appetite and lead to weight gain [13], which is potentially mediated through alterations in the gut microbiome [55]. This is frequently accompanied by impairments in executive function, which facilitates disinhibition and dysregulation of food intake [56], highlighting the importance of the gut–brain axis in psychiatry [57].

Our study has several strengths: First, to our knowledge, this is the first study on subjective views on the diet of people with SMI, in contrast to the biological and quantitative approaches to this topic so far. Further, this study was conducted in different countries where nutrition intervention is offered as routine care in SESLHD (Australia) and other countries where nutrition is usually not part of the treatment of mental health services (Germany and Austria). However, there are some limitations: As this is a qualitative study, there is no general representativeness of our results, partially because of the small sample size. Additionally, several influencing factors, as well as selection bias, could have influenced our results, as people with a higher interest in nutrition or with a high diet-related burden are more likely to participate in these kinds of studies. Further, the focus on physical health and the previous engagement of some participants in a lifestyle intervention at the Sydney site likely impacted results, e.g., when asked whether food impacts mental health, with a high proportion reporting a positive experience. In addition, whether participants followed research on nutrition and mental health on their own accord was not explored. It seems as if the participants receiving the lifestyle intervention at the study site in Sydney were better informed or educated about lifestyle-related health risks. Despite employing a standardized interview schedule and conducting an initial training session, we cannot confirm consistency between interviewers, given the varying levels of experience in qualitative interviews. Importantly, the majority of the participants from all countries were female. Women are known to gain more weight during psychopharmacological therapy and are more likely to be diagnosed with metabolic syndrome when compared to men [58]. However, gender-specific analysis was not possible within the scope of this qualitative study, though gender differences in eating behavior and subjective implications of food are likely [59,60]. Further, we only conducted a thematic analysis, but since nutritional attitudes are already taking shape in the early stages of life, a biographical narrative analysis would be of interest [61].

## 5. Conclusions

In summary, participants described bidirectional relationships between both diet and eating behaviors and their mental and physical health, with negative connotations being more prominent. These qualitative experiences complement and give context to the quantitative data demonstrating poorer diet quality and more frequent disordered/emotional eating behaviors in people living with SMI [12,62]. Although nutritional approaches are already recommended in some treatment guidelines and policy documents for people with SMI, nutrition still needs to be integrated into the biopsychosocial treatment model of mental disorders. Nutrition has not traditionally been a focus or priority for mental health clinicians and has led to limited awareness of the nutritional requirements and challenges of their patients [63]. Increasing the amount of nutrition education for mental health clinicians is required as an initial step. Further, a targeted tool for nutrition risk screening in mental health settings is currently in development [37] and would ideally trigger the nutrition care process. Providing recovery-oriented, specialist-delivered nutrition support may help create a sense of pleasure, enjoyment and well-being during and after eating and increase self-efficacy with food, as well as improve mood, physical health and overall quality of life. 

## Figures and Tables

**Table 1:** Description of study population.

	Total (*n* = 28)	Australia (*n* = 12)	Germany (*n* = 8)	Austria (*n* = 8)
Sex, female; *n* (%)	17 (61%)	7 (58%)	5 (63%)	5 (63%)
Age, in years; m (sd)	43.3 (13.5)	40.0 (15.6)	48.6 (10.7)	43.1 (11.1)
Body mass index (BMI), in kg/m^2^; m (sd)	31.3 (6.4)	31.3 (5.0)	35.6 (7.3)	26.8 (3.7)
Obesity (BMI > 30 kg/m^2^); *n* (%)	16 (57%)	7 (58%)	7 (88%)	2 (25%)
Schizophrenia or related disorders (ICD-10 F2); *n* (%)	16 (57%)	11 (92%)	3 (38%)	2 (25%)
Affective disorders; *n* (%)	18 (64%)	5 (42%)	5 (63%)	6 (75%)

## Data Availability

The original quotes presented in the study are included in the article. Further inquiries can be directed to the corresponding author.

## Supplementary Materials

The following supporting information can be downloaded at: https://www.mdpi.com/article/10.3390/nu14132616/s1, Supplementary File S1.
