# Supplementary material for: Implications of Dietary Intake and Eating Behaviors for People with Serious Mental Illness: A Qualitative Study
Source: Nutrients. 2022 Jun 24;14(13):2616. doi: 10.3390/nu14132616 (PMC9268504; doi:10.3390/nu14132616)
Supplement: Supplementary file 1 [file nutrients-14-02616-s001.zip › nutrients-1772110-SI.pdf]

Supplementary File S1

GUIDE FOR SERVICE USER INTERVIEWS

- Clarify participant number

- Please clarify how long you have been receiving treatment/support for your mental health?

- Clarify the aim for today's interview is exploring factors affecting dietary intake and eating behaviours?

- Clarify diet will refer to food choices giving an example, and eating behaviours will refer to aspects such as appetite (e.g. eat more than others appear to), speed of eating, structure (or lack of structure) of meals over the day, emotional and binge eating, overnight eating,

- How do you rate you're the quality of your dietary intake on a scale of 0-10, 10 being perfect?
    - How happy/satisfied are you with your dietary intake? Please briefly describe why you chose that score.

- How do you rate you're the quality of your eating behaviours on a scale of 0-10, 10 being perfect or satisfied?
    - How happy/satisfied are you with your eating behaviours? Please briefly describe why you chose that score.

- In your experience what challenges or barriers get in the way of you choosing healthier foods?
    - Utilise pre-determined domains as prompts where needed: cravings, motivation, cost, knowledge, preparation skills, food storage challenges.
    - Continue until exhausted options.

- Have you experienced any challenges in terms of eating behaviours?
    - Utilise pre-determined domains as prompts where needed: appetite, binge/emotional eating, eating overnight, planning of meals (including shopping), preparing of meals (cooking, time), financial issues, intolerances.
    - Continue until exhausted options.

- Do you think having mental health challenges or the medications you are prescribed have a role in the development of these challenges and barriers to healthy food choices and eating behaviours? If so, in what way?

- Could you specify any changes that you noticed in your eating behaviours/diet;
  (i) at the onset of mental health challenges or starting medication? and
  (ii) when medications were changed or when new medications were added?

- Do you think what you eat affects your mental health? If so, please explain how and what foods or dietary patterns.

- To what extent have your diet and/or eating behaviours been addressed in health care so far?
    - What do you think could be done to promote the eating behaviour of people with mental illness?

- Thank participant for their involvement and close interview
